# Effectiveness of web-based feedback interventions for people with overweight and obesity: systematic review and network meta-analysis of randomized controlled trials

**DOI:** 10.1186/s40337-021-00432-6

**Published:** 2021-06-26

**Authors:** Carmen Varela, Camila Oda-Montecinos, Ana Andrés, Carmina Saldaña

**Affiliations:** 1 Department of Clinical Psychology and Psychobiology, Section of Personality, Assessment and Treatment, Faculty of Psychology, University of Barcelona, Passeig Vall d’Hebrón, 171, P.C. 08035 Barcelona, Cataluña Spain; 2 Institute of Social Sciences, Universidad de O’Higgins, Rancagua, Chile; 3 Faculty of Psychology, Education and Sport Sciences, Blanquerna, Ramon Llull University, Barcelona, Spain; 4 Institute of Neurosciences, University of Barcelona, Barcelona, Spain

**Keywords:** Obesity, Website, Feedback, Treatment, Network meta-analysis

## Abstract

**Background:**

Web-based delivered interventions have become an innovative option to treat health problems, like obesity. The aim of this systematic review and network meta-analysis was to analyze the effectiveness of web-based behavioral treatments for adults with overweight and obesity. Web-based interventions and comparison interventions (traditional weight control programs) were classified according to the following feedback characteristics: frequency, personalization, and provider (human versus machine).

**Method:**

From the initial 1789 studies, 15 were included in this review. A network meta-analysis was conducted to analyze the efficacy of web-based programs with traditional interventions, considering direct and indirect comparisons. The main outcome was the weight loss mean difference (kg) between baseline and post-treatment. Heterogeneity and consistency assumptions were validated to conduct the network meta-analysis.

**Results:**

Network meta-analysis showed comparisons between different treatment options. The main results were that Intensive Contact Web-based programs were more effective than wait-list (Mean Difference − 1.86 kg; 95% Confidence Interval: − 3.61, − 0.12). Moreover, Intensive Contact Web-based programs were more effective than the other web-based options and self-help traditional interventions. However, the only significant comparison was Intensive Contact Web-based programs versus Guided Self-Help Web-based programs (Mean Difference − 4.31 kg; 95% Confidence Interval: − 5,22, − 3,41). Intensive Contact Web-based programs were the most effective treatment option according the obtained results, achieving the first place in the ranking provided by the network meta-analysis with 98.5% of probabilities.

**Conclusions:**

Intensive Contact Web-based interventions have obtained the first position in the ranking, proving the relevance of frequent, personalized, and professional feedback and their association with a better prognosis for people with overweight and obesity. These results provide relevant information to design more effective treatments for people with overweight and obesity, in a new format especially appropriate for the current situation.

**Supplementary Information:**

The online version contains supplementary material available at 10.1186/s40337-021-00432-6.

## Plain English summary

The number of people with overweight and obesity has tripled worldwide since 1975. Obesity has become a serious problem for public health. For that reason, the design of innovative treatments for people with overweight and obesity is an urgent need. This systematic review and network meta-analysis assesses the effectiveness of innovative interventions, concretely web-based programs for overweight and obesity, comparing them with traditional behavioral treatments and wait-list groups. Both web-based and traditional behavioral treatments were classified according to three feedback characteristics: personalization, frequency, and provider (human versus machine). The literature has suggested that a frequent feedback is an indicator of good prognosis in traditional interventions. The results of this study showed that for web-based interventions, an intensive and personalized feedback provided by a healthcare professional was the treatment option that led to a better prognosis. The second and third option were web-based guided self-hep programs and traditional self-help programs. These interventions empowered the participants, providing more sense of self-efficacy. Web-based minimal-contact programs, web-based self-help programs and wait-list were the last options. An accurate ranking of available treatment options only can be provided by a network meta-analysis, the statistic technique used in this study.

**Supplementary Information:**

The online version contains supplementary material available at 10.1186/s40337-021-00432-6.

## Background

Obesity has become a major public health problem in the recent years [1, 2]. According to the World Health Organization (WHO), in 2016 the prevalence of obesity was 13% worldwide [2], and high-income western countries showed the most remarkable growth of these obesity rates [1, 3]. Obesity has been identified as a risk factor for diseases like hypertension, cancer or diabetes [4], and psychological problems like depression, anxiety or discrimination and bias [5, 6]. These facts highlight the urgent need to investigate new treatments for this resistant problem [7].

The most empirically validated treatments for obesity are traditional behavioral weight control programs [8], achieving greater weight losses than control groups at post-treatment. To measure the effectiveness of a traditional weight control program, the post-treatment weight loss must be equal or more than 5% of the initial weight [9–11]. This kind of interventions are usually delivered by a health care professional and tend to focus on nutrition and exercise [9]. Personalized and frequent feedback, provided by a health care professional, has been identified as a predictor of good prognosis in traditional weight control programs [11–13]. However, most of these interventions present difficulties to achieve the recommended 5% weight loss at post-treatment measure, high adherence rates and long-term weight loss maintenance results [9, 10].

In recent years, web-based interventions for people with overweight and obesity have appeared as a new treatment option [14], as a result of the increase in the use of internet across different generations [15]. New technologies have become an essential tool in daily life [16, 17], and may improve adherence rates [17] due to their easier access, faster feedback, wider reach, and reduced cost. Moreover, web-based interventions promote self-regulation and the perception of self-efficacy. These skills show significant associations with successful results in weight loss programs, social and professional support [11–13]. Web-based professional-supported interventions may cover the identified weaknesses of traditional interventions and incorporate their advantages at the same time. For example, web-based interventions must include personalized and frequent feedback, provided by health care professionals, as a significant component to achieve significant results in terms of effectiveness [12].

Previous reviews show contradictory results about web-based interventions for people with obesity [7, 12, 14, 18–23]. Several studies support that internet-delivered interventions are at least as effective as traditional behavioral treatments in the short term, regarding weight loss [7, 12, 14, 18, 20, 21]. However, online treatments present poorer results compared to traditional interventions where the person is actively involved [22]. Therefore, the focus should be on improving web-based interventions where personalized feedback [11, 12, 23], social support [5, 19, 24, 25], and self-regulation skills [13] are included.

The aim of this systematic review and network meta-analysis (NMA) was to assess the effectiveness of internet-based behavioral treatments for adults with overweight and obesity according to three characteristics of feedback: frequency, personalization and provider. Taking into consideration these variables, comparisons were conducted between delivered (partially or totally) web-based interventions, traditional behavioral treatments and no treatment or wait-list groups.

## Method

The international prospective register for systematic reviews (PROSPERO) accepted the protocol of this systematic review and network meta-analysis on 22nd January 2019, registration number: CRD42019120230 [26]. This proposal follows the guideline of Preferred Reporting Items for Systematic Reviews incorporating Network Meta-analysis (PRISMA-NMA) [27].

### Search strategy

The electronic databases searched were PubMed, Cochrane Central Register of Controlled Trials (CENTRAL), Web of Science (WoS), Scopus and PsycInfo. The search was closed on 6th February 2019. Two updates of the search were conducted, the first one on 5th September 2019 and the second one on 10th June 2020.

Three were the limitations imposed on the search: the study design had to be randomized controlled trials (RCTs); the language had to be English and Spanish; and the population had to be adults between 18 and 65 years. There were no limits regarding the year of publication.

According to the different databases search formats, the combination of keywords was the same in all of them: (obesity OR obese OR overweight) AND ((“randomized-controlled trial” OR “randomized controlled trial” OR “controlled clinical trial” OR “clinical trial as a topic”) OR (randomly OR trial OR randomized OR placebo)) AND (internet OR web OR website OR computer OR online) AND (behavior OR behaviour OR behavioral OR behavioural OR cognitive OR cognitive-behavioral OR cognitive-behavioural OR “weight loss” OR “weight management” OR “weight maintenance” OR program OR intervention OR treatment).

### Study selection

A total of 1948 articles were identified and imported into Rayyan, a web application designed to work to systematic reviews. However, 159 articles presented an error during the importation process because they were damages. Rayyan removed them automatically. Thus, 1789 articles were available to begin the screening process. After removing the duplicates, two independent reviewers screened titles and abstracts according to the pre-specified criteria. Selected articles were full-text evaluated independently by the same two reviewers. References of full-text selected articles were searched manually. Finally, disparities were solved through discussion between the two reviewers and a third reviewer.

In the first update, 5th September 2019, 39 new articles were identified and revised one by one. However, none of them accomplished the inclusion criteria. On the 10th June 2020, 430 new articles were identified and revised one by one again. None of them accomplished the required inclusion criteria.

### Inclusion and exclusion criteria

The studies had to accomplish the following criteria to be included in the review:

#### Population

Adult population (18–65 years) with overweight and obesity, with a body mass index (BMI) between 25 and 39.9 kg/m^2^. People with a BMI > 40 kg/m^2^ were excluded because these types of obesity are usually associated with physical complications which might need pharmacological treatment or bariatric surgery [2].

#### Intervention

Web-based behavioral programs for overweight and obesity. Programs delivered only by a website or website combined with other technological devices (e.g. internet and a mobile application), and which belong to the classification of behavioral change programs. The reason to choose web-based interventions only and exclude programs delivered through other digital devices was because websites are currently the most validated tools to administrate a psychological intervention using new technologies. It is necessary to prove their effectiveness in the first place, as there are many web-based interventions available. For that reason, we decided to focus on them for this systematic review. The interventions were classified by intensity of contact (self-help, guided self-help, minimal contact, or intensive contact). However, traditionally, the intensity of feedback was studied based on its frequency and it was demonstrated that personalized feedback is a relevant variable to consider in web-based interventions [12, 28]. It is important to clarify what it is understood as personalized feedback, because automatic messages based on registered data were often considered personalized feedback [29]. However, in psychology, the feedback provided to a client should consider the individual characteristics of each person and, healthcare professionals (e.g. psychologist, nurse or physician) develop this task properly. For those reasons, it is important to consider the feedback provider, machine or human, to make the classification [12]. In order to be as feasible as possible to real contexts, in this study, the classification considered these three factors: frequency, feedback personalization, and the feedback provider [12, 23]. Therefore, based on these principles the final classification was as follows:

- Self-help Website (SH-W): There was no feedback during treatment. Only face-to-face meetings took place at the beginning and at the end of the treatment. Therefore, there was no need to consider the feedback provider in this category.
- Guided Self-Help Website (GSH-W): Considering the frequency, in this category the proportion of feedback was sporadic depending on the length of the treatment. For example, only took place once in the middle of the intervention (online feedback or face-to-face meeting). If the feedback provider was a machine, messages with objective data or graphs but without personalized information belongs to this category, no matter the frequency of these messages. This feedback was considered guided self-help, because the information could help the participants, but it was not adapted to their specific needs.
- Minimal Contact Website (MC-W): Feedback was provided, at least, once a month (online or face-to-face meeting) and personalized. If the feedback provider was a machine, the message must contain objective data and information to address treatment goals at least once a month. However, these messages were not adapted to the specific needs of the participants.
- Intensive Contact Website (IC-W): Feedback was provided, at least, once a week (online or face-to-face meeting) and personalized, only health care professionals as feedback providers.

#### Comparator

Any traditional behavioral treatment designed for people with overweight and obesity (e.g. face-to-face, group therapy, primary care). Control groups and wait-list were considered for inclusion. Treatments with drugs or using new technologies or digital devices but no internet as the main source were excluded. Comparators were also classified by different levels of intensity of contact with the health care professionals. In this case, the provider was always a person. For that reason, the classification was, mainly, made based on frequency.

- Self-help (SH)*:* No feedback during the treatment. Only face-to-face meetings at the beginning and at the end of the treatment.
- Guided Self-Help (GSH): Sporadic proportion of feedback, face-to-face, depending on the length of the treatment. For example, in the middle of the intervention.
- Minimal Contact (MC)*:* At least once a month face-to-face proportion of feedback.
- Intensive Contact (IC)*:* At least once a week face-to-face proportion of feedback.

#### Outcome

Main outcome is body weight change (kg), mean difference from baseline to post-intervention. Secondary outcomes are maintenance of weight loss, mean difference from baseline to follow-up and dropout rates.

#### Study design

Randomized controlled trials.

### Data extraction

Data extraction was done by two independent reviewers (CV and CO) and validated by a third reviewer (CS). The extracted information was: i) characteristics of the study: authorship, year, country, sample size, setting and design; ii) characteristics of the population: gender, age, ethnicity, weight, BMI, no presence of comorbid diseases; iii) characteristics of the interventions and comparators: intensity of contact with a health care professional, feedback provider, behavioral programs (nutrition, physical activity and self-records), length of the intervention, length of the follow-up; iv) characteristics of the outcomes: weight loss post-intervention, weight loss maintenance, drop-out rates.

### Quality assessment

Two independent reviewers assessed the studies considered for inclusion in order to ensure the methodological quality of the study, according to the criterion of Cochrane Collaboration Handbook [30]. Cochrane’s tool provided 7 quality domains with specific criteria to decide between high, low or unclear risk of bias. The assessed domains were sequence generation, allocation concealment, blinding of participants/personnel, blinding of outcome assessment, incomplete outcome data, selective outcome reporting and other sources of bias [30].

### Analysis

STATA/IC 14.2 was used to perform the NMA in a frequentist framework. For the main outcome, the preferred measure was the mean difference of weight loss in kg between baseline and post-intervention, a continuous variable. Mean difference was also used as the effect measure [31].

Network meta-analysis is a popular statistical technique, which combines evidence on multiple trials comparing multiple treatments [31, 32]. The special characteristic of this type of analysis is the integration of direct evidence, direct comparisons among the studies, and the indirect evidence, comparison of two treatments via a common comparator [33]. A contribution plot was conducted to identify the most influential direct comparisons for the entire network and for each network estimated [32, 33].

Homogeneity and consistency assumptions are essential to evaluate the validity of the NMA [34]. A conventional pairwise meta-analysis was conducted to assess overall heterogeneity [35]. Considering the results of this meta-analysis, a subgroup meta-analysis stratified by frequency and personalized feedback have been performed with STATA and RevMan5, using the inverse variance weighted random effects model [34]. To measure heterogeneity, the established parameters are the following for the *I*^*2*^ statistic: 25% low heterogeneity, 50% moderate heterogeneity and 75% high heterogeneity [36]. The calculation of heterogeneity through a pairwise meta-analysis is the current way to know this index when a network meta-analysis has been conducted. However, the multivariate heterogeneity measures developed for multivariate meta-analysis are not available yet for NMA [33]. For that reason, in network meta-analysis, it is common to assume a common heterogeneity variance across all pairwise comparisons [31, 33].

Consistency assumption sustains that the relationship between direct and indirect sources of evidence for a single comparison should be consistent [37]. Wald test for inconsistency was applied to ensure the consistency assumption [31]. Local consistency was assessed with the node splitting model for each treatment contrast and with an inconsistency plot to measure the inconsistency factor, which is the absolute difference between direct and indirect effects for the same comparison, for each closed loop in the NMA [31–33]. Finally, separated meta-regressions were conducted for the following variables: age, length, initial weight, risk of bias and sample size.

For the secondary outcomes, pairwise meta-analysis was carried out to analyze long-term weight loss results in the articles with follow-up measures. Besides, we reported dropout rates as percentages for the different interventions.

## Results

The use of acronyms is necessary in this review because there are many intervention arms. To facilitate the understanding of this section, there is a reminder of the acronyms used through this study: Intensive Contact Web-based (IC-W), Minimal Contact Web-based (MC-W), Guided Self-Help Web-based (GSH-W), Self-Help Web-based (SH-W), Self-Help (SH) and Wait-list.

**Fig. 1:**
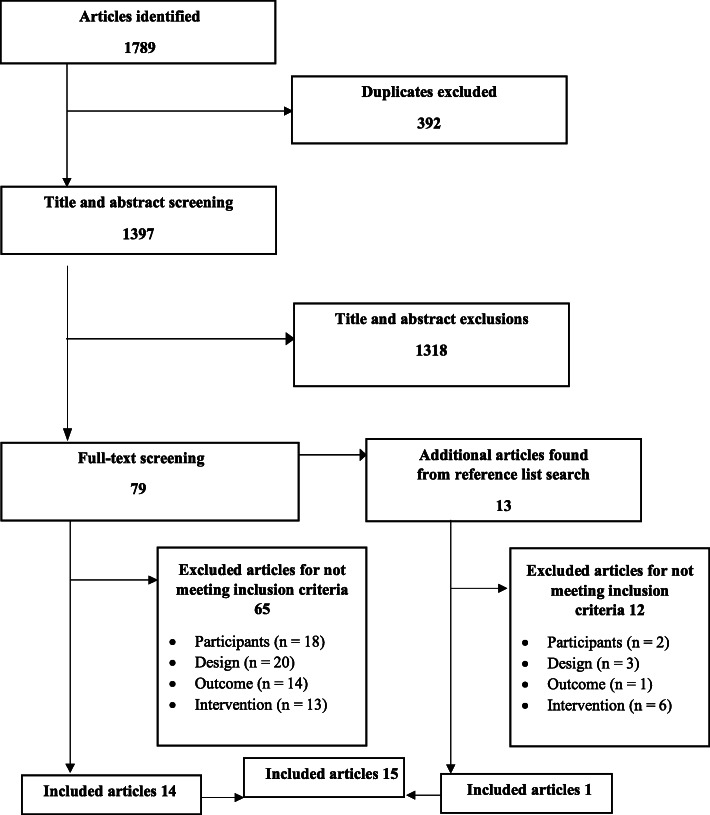
Flow diagram of study selection

The number of articles identified was 1789. After removing duplicates and the screening of titles and abstracts by two independent reviewers, 79 studies were selected for full-text screening. Reference lists of these articles were searched, and 13 studies were included in the full-text screening. Finally, 15 articles [38–52] met the inclusion criteria and were included in the review (Fig. 1).

The overall quality assessment of the included studies identified 9 with low risk of bias, 5 with unclear risk of bias and only 1 trial with high risk of bias. The detailed risk of bias assessment is available in additional file Figure A.1.

### Description of included studies

Table 1 summarizes the main characteristics of included studies. The 15 articles were published from 2001 to 2017 and were developed in Australia (*n* = 8) and the United States (*n* = 7). The total number of participants was 2426, 48.4% of them being women (*n* = 1173) and having a mean age of 44.8 (SD = 0.9) years. Following the inclusion criteria, the whole sample presented overweight and obesity, with a BMI ranging from 29 to 33.9 kg/m^2^. All the studies presented at least one intervention arm delivered via online, 24 web-based intervention arms were identified and 12 traditional comparators. Interventions were behavioral programs with at least these three treatment areas: nutrition, physical activity and records, both web-based programs and traditional active comparators. The median length treatment was 18.3 weeks (range 12–48 weeks), only five studies presented follow-up measures after the end of the intervention.

**Table 1:** Characteristics of included studies

Study	Mean age (SD)	Mean BMI (SD)	% of females	Intervention(s)	Comparator(s)	Intensity of contact	Feedback provider	Intervention length	Follow-up
**Blomfield et al. 2013** [38] **Australia**	47.5 (11.0)	32.7 (3.5)	0	SHED-IT online (*n* = 53)	SHED-IT Program materials (*n* = 54) Wait-list (*n* = 52)	IC (SHED-IT WBE) SH (SHED-IT Program materials)	Human (SHED-IT)	12 weeks	24 weeks
**Chambliss et al.** **2011** [39] **USA**	45.0 (10.3)	30.5	83	WBB (*n* = 45) WBE (*n* = 45)	Wait-list (*n* = 30)	GSH (WBB) MC (WBE)	Human (Both conditions)	12 Weeks	–
**Collins et al. 2012** [41] **Australia**	42.0 (10.2)	32.3 (4.0)	58	WBB (*n* = 99) WBE (*n* = 106)	Wait-list (*n* = 104)	SH (WBB) MC (WBE)	Machine (WBE)	12 weeks	–
**Collins et al. 2013** [40] **Australia**	41.9 (10.2)	32.2 (3.9)	58.5	WBB (*n* = 143) WBE (*n* = 158)	Comparison between intervention groups	SH (WBB) MC (WBE)	Machine (WBE)	24 weeks	–
**Gabriele et al. 2010** [42] **USA**	45.4 (8.7)	32.1 (4.3)	83.7	Minimal E-Coach Support (*n* = 34) Directive E-Coach Support (*n* = 35) Non-Directive E-Coach Support (*n* = 35)	Comparison between intervention groups	GSH (Minimal E-Coach Support) MC (Non-Directive E-Coach Support) IC (Directive E-Coach Support)	Human (All conditions)	12 weeks	–
**Gold et al. 2007** **USA** [43]	47.7 (10.3)	32.4 (4.1)	81.5	eDiets (*n* = 62) VTrim (*n* = 62)	Comparison between intervention groups	MC (eDiets) IC (VTrim)	Machine (eDiets) Human (VTrim)	24 weeks	48 weeks
**Hunter et al. 2008** [44] **USA**	34.0 (7.3)	29.4 (3.0)	50.2	BIT + LEARN program (*n* = 224)	Usual Care (*n* = 222)	IC (BIT + LEARN) SH (Usual Care)	Human (BIT + L.EARN)	24 weeks	–
**Morgan et al. 2009** [47] **Australia**	35.9 (11.1)	30.6 (2.8)	0	SHED-IT (*n* = 34)	Information (*n* = 31)	IC (SHED-IT) SH (Information)	Human (SHED-IT)	12 weeks	24 weeks
**Morgan et al. 2010** [48] **Australia**	35.9 (11.1)	30.6 (2.8)	0	SHED-IT (*n* = 34)	Information (*n* = 31)	IC (SHED-IT) SH (Information)	Human (SHED-IT)	12 weeks	48 weeks
**Morgan et al. 2011** [46] **Australia**	44.4 (8.6)	30.5 (3.6)	0	POWER (*n* = 65)	Wait-list (*n* = 45)	IC (POWER)	Human (POWER)	14 weeks	–
**Morgan et al. 2013** [45] **Australia**	47.5 (11.0)	32.7 (3.5)	0	SHED-IT (*n* = 53)	SHED-IT Program materials (*n* = 54) Wait-list (*n* = 52)	IC (SHED-IT) SH (SHED-IT Program materials)	Human (SHED-IT)	12 weeks	24 weeks
**O’Brien et al. 2014** [49] **Australia**	41.6 (10.2)	32.3 (3.9)	58.5	WBB (*n* = 94) WBE (*n* = 98)	Wait-list (*n* = 97)	SH (WBB) MC (WBE)	Machine (WBE)	12 weeks	–
**Tate et al. 2001** [50] **USA**	40.9 (10.6)	29.0 (3.0)	89	IE (*n* = 45) IBT (*n* = 46)	Comparison between intervention groups	MC (IE) IC (IBT)	Human (Both conditions)	24 weeks	–
**Thomas et al. 2017** [51] **USA**	55.0 (11.5)	33.9 (3.7)	77.5	WWO (*n* = 94) WWO + AL (*n* = 91)	Newsletters (*n* = 86)	SH (WWO) GSH (WWO + AL) SH (Newsletters)	Machine (WWO + AL)	48 weeks	–
**Womble et al. 2004** [52] **USA**	43.7 (10.2)	33.5 (3.1)	100	eDiets (*n* = 23)	LEARN program (*n* = 24)	GSH (eDiets) SH (LEARN)	Human (eDiets)	48 weeks	–

*BIT* Behavioral Internet Therapy, *GSH* Guided Self-Help, *IC* Intensive contact, *IBT* Internet Behavior Therapy, *IE* Internet Education, *LEARN* Lifestyle Exercise Attitudes Relationships Nutrition, *MC* Minimal Contact, *POWER* Preventing Obesity Without Eating Like a Rabbit, *SH* Self-Help, *SHED-IT* Self-Help Exercise and Diet using Internet Technology^a^, *WBB* Web-Based Basic, *WBE* Web-Based Enhanced

^a^Regardless of the SHED-IT program means Self-Help, in this study have been classified as intensive contact because of the proportion of weekly-personalized feedback the four first weeks

**Fig. 2:**
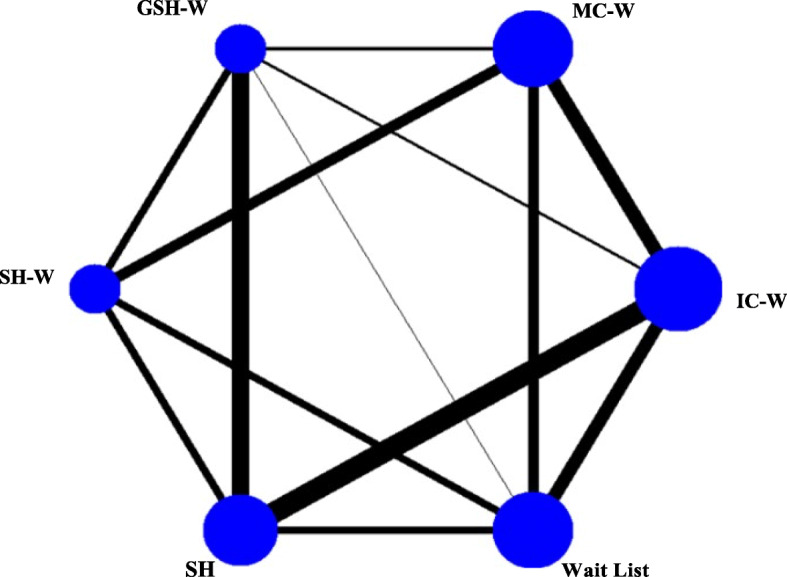
Network diagram of available comparisons. The size of each edge is proportional to the number of studies available for each comparison. GSH-W, Guided Self-Help Web; IC –W, Intensive Contact Web; MC-W, Minimal Contact Web; SH, Self-Help; SH-W, Self-Help Web

The participants of the 15 trials were randomized to 36 intervention arms relevant to this review. Based on our frequency and personalized feedback classification, interventions were allocated to 6 different categories (Fig. 2). In this review, we identified four categories for web-based interventions through the included studies: intensive contact in 9 intervention arms, minimal contact in 7 arms, guided self-help in 4 arms and self-help in 4 arms. However, the selected studies for our review only presented two categories for the traditional comparators, self-help in 7 arms and 6 arms for wait-list. Table 1 shows the feedback provider for each intervention arm, which could be a health care professional or a machine.

A contribution plot (see additional file Figure A.2) was generated to know the influence of each direct comparison to the estimation of each network meta-analytic summary effect by a different weight. In this case, 13 direct comparisons were identified as relevant in the network, the most influential direct comparison for the entire network with a contribution of 12.2% was IC-W versus SH. Two indirect comparisons were identified Intensive Contact Web-based versus Self-Help Web-based and Minimal Contact Web-based versus Self-Help. The contribution plot is available in the supplementary material.

### Pairwise meta-analysis results

Wait-list groups showed poorer results in terms of efficacy than web-based intervention groups in all feedback categories: IC-W versus Wait-list (Mean Difference (MD) -4.32; 95% CI: − 5.08, − 3.57), MC-W versus Wait-list (MD -3.23; 95% CI: − 3.80, − 2.66), GSH-W versus Wait-list (MD -3.02; 95% CI: − 4.28, − 1.76) and SH-W versus Wait-list (MD -2.55; 95% CI: − 3.12, − 1.97) (see additional file Figures A.3-A.6).

Similar results were obtained for comparisons of web-based interventions versus self-help programs, IC-W versus SH (MD -1.79; 95% CI: − 2.33, − 1.24), SH-W versus SH (MD -0.80; 95% CI: − 2.21, − 0.61). GSH-W versus SH was the only comparison in favor of SH instead of the web-based option (MD 0.55; 95% CI: − 0.66, 1.77) (see additional file Figures A.7-A.9). However, the 95% CI of the GSH-W and SH-W comparisons versus SH, showed that the effect was not significant. MC-W versus SH was not tested in any of the selected studies, and therefore was not included in the further meta-analysis.

In order to identify which web-based program according to frequency and personalized feedback was more effective, comparisons between the different categories were conducted (see additional file Figures A.10-A.14). Significant results were obtained for the following comparisons IC-W versus MC-W (MD -1.28; 95% CI: − 3.53, − 0.93) and MC-W versus SH-W (MD -0.74; 95% CI: − 1.32, − 0.16).

### Network meta-analysis assumptions: heterogeneity and consistency

Regarding heterogeneity assumption, a pairwise meta-analysis was conducted. The overall heterogeneity index was high *I*^*2*^ = 80.2%. However, the aim of this review was to study differences between treatments attending the frequency and personalization of feedback. Reviewing the results of the meta-analysis the classification variable has been identified as the cause of the heterogeneity because, for example, the results with intensive contact should differ from the results of self-help. For this reason, a stratified meta-analysis by the classification of the interventions by frequency and personalized feedback was conducted. The results of subgroup pairwise meta-analysis are available in the supplemental material (Figures S3-S14), all comparisons presented low heterogeneity *I*^*2*^ = 0%, except GSH-W versus SH, which showed a heterogeneity of *I*^*2*^ = 77%. As in NMA heterogeneity is expected, to ensure the homogeneity assumption, common heterogeneity variance is assumed across pairwise comparisons [31, 33].

To ensure the presence of consistency, Wald test was conducted and did not identify the presence of inconsistency (*X*^*2*^ = 13.2; *p* = 0.21). Local inconsistency for each treatment contrast was calculated; the results supported consistency because no *p* values were statistically significant (Table 2).

**Table 2:** Inconsistency test between direct and indirect treatment comparisons

Side	Direct		Indirect		Difference		p > z
	Coefficient	SE	Coefficient	SE	Coefficient	SE	
**IC-W versus Wait-list**	4.318	0.377	5.064	0.461	−0.746	0.612	0.223
**IC-W versus MC-W**	2.210	0.559	1.234	0.402	0.976	0.688	0.156
**IC-W versus GSH-W**	1.296	0.893	2.149	0.468	−0.853	1.011	0.399
**IC-W versus SH**	1.791	0.280	2.063	0.704	−0.272	0.759	0.720
**MC-W versus Wait-list**	3.215	0.286	2.524	0.534	0.690	0.617	0.263
**MC-W versus GSH-W**	−0.098	0.536	0.961	0.571	−1.059	0.782	0.175
**MC-W versus SH-W**	0.739	0.296	−0.930	0.677	1.669	0.744	0.025
**GSH-W versus Wait-list**	2.952	0.638	2.502	0.468	0.451	0.780	0.564
**GSH-W versus SH-W**	−0.532	0.699	0.367	0.494	−0.899	0.854	0.292
**GSH-W versus SH**	−0.575	0.621	0.210	0.549	−0.784	0.830	0.344
**SH-W versus Wait-list**	2.598	0.292	2.564	0.505	0.034	0.584	0.954
**SH-W versus SH**	0.807	0.717	−0.526	0.405	1.333	0.824	0.106
**SH versus Wait-list**	2.732	0.426	2.854	0.430	−0.121	0.614	0.843

*GSH-W* Guided Self-Help Web, *IC-W* Intensive Contact Web, *MC-W* Minimal Contact Web, *SE* Standard Error, *SH* Self-Help, *SH-W* Self-Help Web

Consistency assumption was confirmed with the generation of an inconsistency plot to assess the inconsistency factor for each closed loop in the NMA. In 13 loops, only one presented significant inconsistency (IC-W, MC-W, SH-W, SH; IF = 1.96; 95% CI: 0.01–3.92; *p* = 0.049). However, this result was not relevant enough to reject the consistency assumption. Besides, the heterogeneity specific for each loop was low, except for one loop (GSH-W, SH-W, Wait-list; *r*^*2*^ = 0.794), this comparison was the same with high heterogeneity in the conventional meta-analysis (see additional file Figure. A.15).

### Network meta-analysis results

**Fig. 3:**
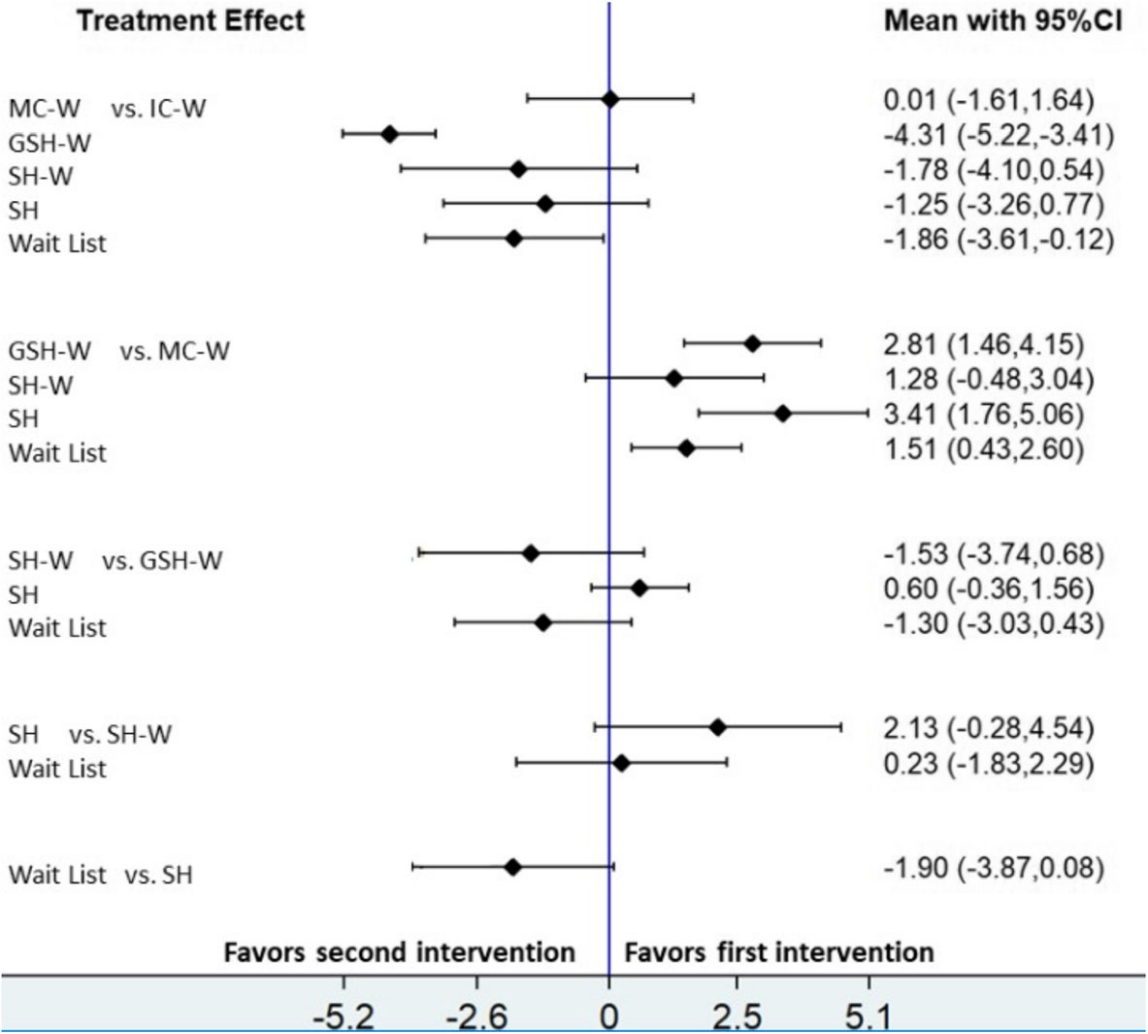
Estimates (mean difference and 95% credible intervals) from network meta- analysis for the difference of weight lost. CI, Confidence Interval; GSH-W, Guided Self-Help Web; IC –W, Intensive Contact Web; MC-W, Minimal Contact Web; SH, Self-Help; SH-W, Self-Help Web

Figure 3 shows the results for the network meta-analysis considering direct and indirect comparisons (Fig. 3 and Table A.1 in the additional file). Comparing with wait-list only IC-W (MD -1.86; 95% CI: − 3.61, − 0.12) and MC-W (MD 1.51; 95% CI: 0.43, 2.60) obtained significant differences. However, MC-W was less effective than wait-list; these results could be explained inspecting the funnel plot (see additional file Figure A.16). The asymmetry observed in the funnel plot suggested that small trials tend to exaggerate the effectiveness of wait-list compared with MC-W.

Comparing web-based programs with SH, the last one was more effective than the web-based interventions, with the exception of IC-W (MD -1.25; 95% CI: − 3.26, 0.77). The only significant comparison in this case was with MC-W (MD 3.41; 95% CI: 1.76, 5.06) (Fig. 3).

Regarding the comparisons of web-based programs with each other, significant results were obtained for IC-W versus GSH-W (MD -4.31; 95% CI: − 5.22, − 3.41) and MC-W versus GSH-W (MD 2.81; 95% CI: 1.46, 4.15). In general, results should be interpreted carefully; the visual inspection of the funnel plot (Supplementary material, Fig A.16) showed exaggerated effects for self-help and wait-list comparing with some active comparators, especially MC-W, where for example the comparison MC-W versus SH is only based on indirect comparisons (Fig. 3).

**Fig. 4:**
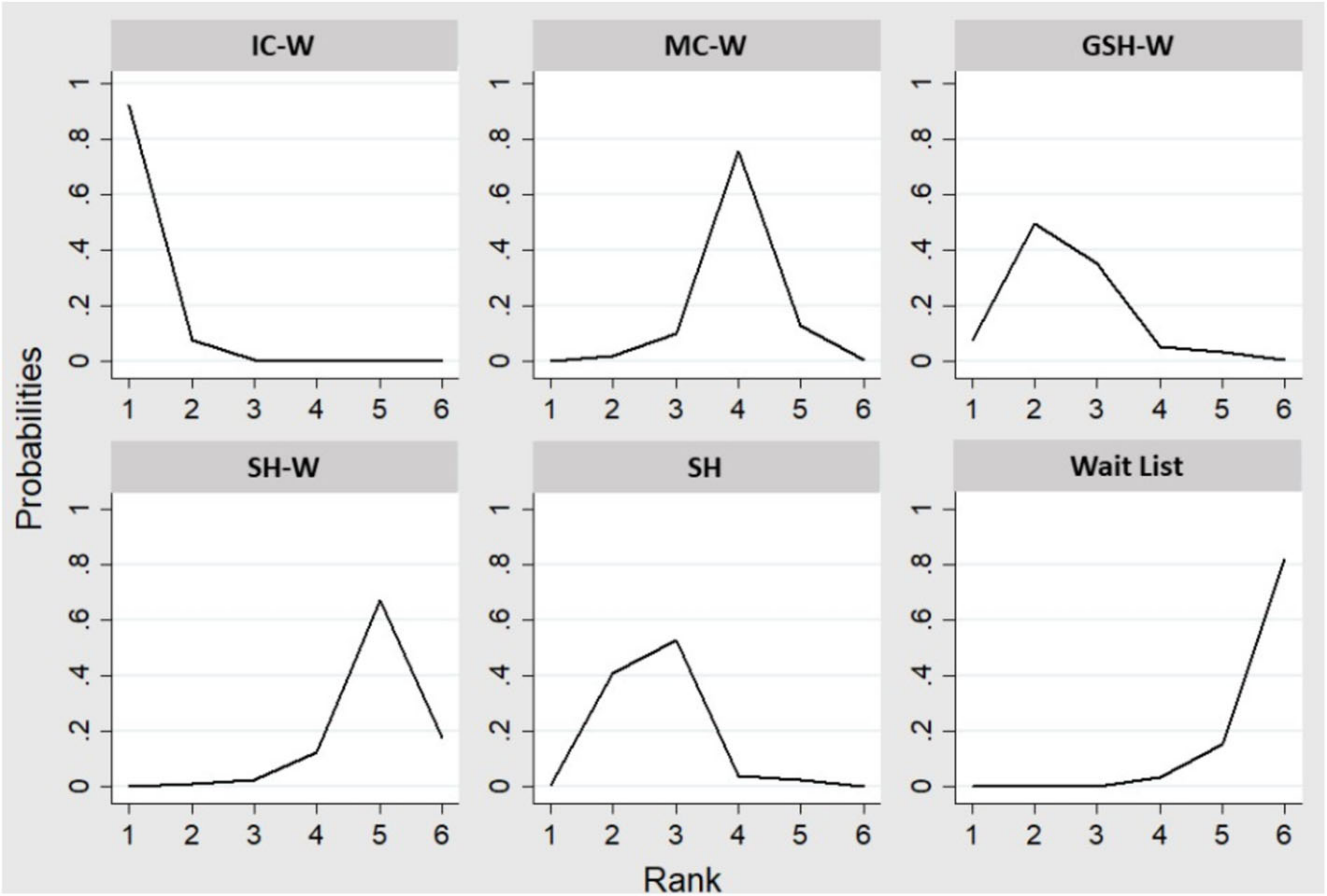
Estimated probabilities of each treatment in the rank. GSH-W, Guided Self-Help Web; IC –W, Intensive Contact Web; MC-W, Minimal Contact Web; SH, Self-Help; SH- W, Self-Help Web

Finally, we presented the rank order of treatments to show the most effective treatment evaluated (Fig. 4). IC-W interventions had approximately 98.5% of probabilities to be the treatment to achieve a greater post-treatment weight loss. GHS-W and SH have appeared in second and third positions with 70.5 and 66.6%, respectively. Finally, MC-W was the fourth option with 40% and, SH-W and wait-list the last ones with 20.2 and 4.2%, respectively.

Separate meta-regressions were carried out to test the effect of age, sample size, risk of bias, length and initial weight. Length was the only variable to show a significant result (*p* = 0.014).

### Long-term weight loss results and dropout

There were only five articles with long-term weight loss results [38, 43, 45, 47, 48]. Three of these studies made the follow-up at 24 weeks from the beginning of the program [38, 45, 48]. In the other two the follow-up was conducted 48 weeks from the beginning of the intervention [43, 47].

Available comparisons in these studies were IC-W versus SH, IC-W versus Wait-list and IC-W versus MC-W. It was possible to conduct a simple pairwise meta-analysis because three studies [38, 45, 48] shared the same design, IC-W versus SH and length of follow-up, 24 weeks (MD -1.60; 95% CI: − 2.81, − 0.38). We observed a small increase of weight comparing with post-treatment measures (MD -1.79; 95% CI: − 2.33, − 1.24). Regarding IC-W versus Wait-list, two studies presented the same design and length, 24 weeks. In this case, we observed a small decrease (MD -4.65; 95% CI: − 5.79, − 3.50) comparing with the post-treatment measures (MD -4.32; 95% CI: − 5.08, − 3.57).

There were not relevant differences in dropout between active conditions (IC-W 19.9%; MC-W 14.4%; GSH-W 20.2%; SH-W 14.9% and SH 16.3%). Wait-list was the condition with the lowest rate of dropout 7.8%. We separate web-based programs from self-help and wait-list to analyze the dropout rates. In total, web-based programs obtained 17.3% of dropout compared with 13.1% in the other conditions.

## Discussion

To the best of our knowledge, this is the first network meta-analysis about the relevance of feedback in web-based delivered treatments for people with obesity. The use of this innovative statistical technique is interesting because it the available data are analyzed in a more comprehensive way than traditional meta-analysis. Findings from this systematic review suggest that web-based interventions with frequent and personalized feedback, provided by a health care professional, are the most effective option for people with obesity.

The measure of efficacy was the mean difference of weight loss pre- and post- treatment. Intensive Contact Web-based are the interventions reporting a greater weight loss comparing with the other conditions, especially IC-W versus Wait-list. To know the treatment options ordered by their effectiveness in terms of weight loss, network meta-analysis provides an innovative tool, a ranking of the included treatments in the systematic review. In this case, IC-W interventions achieve the first place in this ranking with a 98.5% of probabilities and wait-list the last with 4.2%. Previous investigations support the results of this study, where people with obesity receiving personalized feedback provided by a health care professional are more likely to achieve significant weight losses than no feedback groups [12].

Therefore, the presence of a heath care professional was identified as an element of good prognosis [11, 12, 53]. However, self-regulation skills were also recognized as a feasible predictor of success in weight control-programs [13]. In our review, Guided Self-Help Web-based and Self-Help are the second and the third interventions in the ranking. These kinds of programs promote the development of active coping strategies, and the feeling of being responsible for the achievements [54, 55]. Moreover, these GSH-W and SH interventions might be helpful for people with obesity to avoid discriminatory behaviors of health care professionals [56].

However, Self-Help Web-based is the fifth treatment option in the ranking, just ahead the last one, wait-list. GSH-W might be more effective than SH-W because, apart from promoting self-regulation skills, this treatment option presents personalized feedback providing security to the participants [12, 23]. Regarding the greater weight loss observed in SH versus SH-W, the novelty component could be the explanation variable. These two treatment options are very similar and there in no feedback in any of them. Considering this information and the mean age of our sample, 44.8 (SD = 0.9) years, in the absence of feedback, this generation could feel more comfortable with traditional programs rather online interventions [22]. In addition, people might prefer to choose an option whose efficacy was proved in several occasions. For example, one of the included studies used the LEARN program in the SH intervention arm [11], designed almost 20 years ago [57].

The most controversial group is Minimal Contact Web-based interventions. This intervention arm, jointly with GSH-W, include the possibility of a machine as feedback provider. In the network meta-analysis, there is no significant effectiveness differences between IC-W versus MC-W interventions. However, according network meta-analysis results MC-W is less effective than the rest of intervention arms. Moreover, in the ranking it MC-W is in the fourth position. The contribution of the direct comparisons of IC-W versus MC-W estimated for the network meta-analysis was quite high 12.8%. Examining the three involved studies in this comparison [42, 43, 50], two of them present a health care professional providing feedback in both options [42, 50]. For those reasons, there are no relevant differences between them. However, further investigation is necessary to contrast this information.

The following characteristics were identified as relevant in successful web-based interventions for people with obesity: the presence of a health care professional providing frequent feedback, the development of self-regulation skills or the election of self-help programs to avoid judgmental environments [12, 13, 23, 54–56]. For those reasons, it seems that MC-W group is the most controversial treatment option and, it is more difficult to make a statement of its effectiveness. In Minimal Contact Web-based intervention, the motivational and support component of Intensive Contact Web-based intervention is not present enough [24, 58, 59]. Moreover, the presence of a machine providing the feedback, could decrease the feeling of receiving a personalized treatment, even the automated message was based on personal information [12, 23].

Therefore, the presence of personalized and frequent feedback, provided by a health care professional, may be an indicator of good prognosis for people with obesity involved in internet-delivered interventions. This review is a first approximation to prove the relevance of feedback in web-based programs, and the main aspects to design this kind of interventions considering these results are frequency, personalization and feedback provider. Moreover, to ensure that participants feel the support of a health care professional in web-based interventions, this review reveals the importance to highlight the presence of feedback. For example, in MC-W this presence is confusing. This kind of interventions may not present the advantages of IC-W and GHS-W/SH treatments. The ranking tool is especially informative to make choices based on it, and its interpretation is useful for both health care professionals and participants.

The results for long-term weight losses in web-based programs are less relevant, due to the small number of studies with follow-up data [38, 43, 45, 47, 48]. The available comparisons are IC-W versus SH and IC-W versus wait-list. The first one suggests a small increase of weight loss regarding short-term results, and the second one present a small decrease compared with short-term results. However, the differences are too small to make assumptions. Further investigation is needed about long-term results in web-based programs for people with obesity. Dropout rates are quite similar between interventions, comparing internet-delivered treatments with SH and wait-list jointly, the difference is only a 4.2% bigger for web-based programs than traditional options.

This systematic review and network meta-analysis also has some limitations. The automatic removal made by Rayyan of 159 articles. The small number of included studies. The classification of interventions was too specific, attending to three criteria (frequency, personalization and provider of feedback). Regarding personalization criteria, previous researches and clinical practice proved its relevance. However, it is important to mention that a certain extent of subjectivity is normally present on personalization. Some of the studies present samples composed only by women or men with a high mean age [38, 45–48, 52]. Therefore, sociodemographic criteria might affect the results because a positive association was observed between age and weight. More research is needed into the role of automated feedback, provided by a machine, because previous researches were mainly focused on feedback provided by health care professionals. Network meta-analysis is a complex technique and results should be interpreted carefully. At the same time, this statistical approach provides very useful information and is more comprehensive than conventional meta-analysis. Further investigative research should be driven to supply these limitations.

To conclude Intensive Contact Web-based is the first treatment option according to the rank results of this review, supporting the relevance of frequent and personalized feedback, provided by a health care professional. This option provides motivation and support to the participant. However, the general conclusion of the research is to remark the importance to consider feedback designing web-based programs for people with obesity. Personalization is a difficult variable to measure, but its presence is determinant for the success of any weight control program [23]. This review present current clinical implications, because provides a tool to choose the most effective option. Moreover, this review is the first step to drive more researches related with the topic and this information could be useful to design new web-based programs for people with obesity in the future. The importance of the personalized feedback and the preference of a health care professional, instead of a machine, reveals the importance of focusing the design of the treatment on the person who is suffering the problem and not on the problem itself.

## Conclusions

Network meta-analysis has been a helpful tool to analyze the efficacy of web-based programs for people with obesity. Intensive Contact Web-based interventions have obtained the first position in the ranking, proving the relevance of personalized feedback. Further investigation is needed, but these results are a first step in the design of the internet-delivered treatments, an innovative field of research. Moreover, the results of this review support the importance of focusing on the person who suffers obesity instead of the diagnosis or symptoms of this problem.

## Supplementary Information

**Additional file 1: Figure A.1.** Graphic for risk of bias. **Figure A.2.** Contribution direct comparisons table to the network meta-analysis. **Figure A.3.** Intensive Contact Web (IC-W) vs. Wait list. **Figure A.4.** Minimal Contact Web (MC-W) vs. Wait list. **Figure A.5.** Guided Self-Help Web (GSH-W) vs. Wait List. **Figure A.6.** Self-Help Web (SH-W) vs. Wait List. **Figure A.7.** IC-W vs. Self-Help (SH). **Figure A.8.** GSH-W vs. SH. **Figure A.9.** SH-W vs. SH. **Figure A.10.** IC-W vs. MC-W. **Figure A.11.** IC-W vs. GSH-W. Figure **A.12.** MC-W vs. GSH-W. **Figure A.13.** MC-W vs. SH-W. **Figure A.14.** GSH-W vs. SH-W. **Figure A.15.** Inconsistency plot. **Table A.1.** Pairwise treatment comparisons for network meta-analysis for weight loss. **Figure A.16.** Comparison adjusted funnel-plot.

## Data Availability

Not applicable.
