# Supplementary material for: Effectiveness of web-based feedback interventions for people with overweight and obesity: systematic review and network meta-analysis of randomized controlled trials
Source: J Eat Disord. 2021 Jun 26;9:75. doi: 10.1186/s40337-021-00432-6 (PMC8234624; doi:10.1186/s40337-021-00432-6)
Supplement: Supplementary file 1 — Additional file 1: Figure A.1. Graphic for risk of bias. Figure A.2. Contribution direct comparisons table to the network meta-analysis. Figure A.3. Intensive Contact Web (IC-W) vs. Wait list. Figure A.4. Minimal Contact Web (MC-W) vs. Wait list. Figure A.5. Guided Self-Help Web (GSH-W) vs. Wait List. Figure A.6. Self-Help Web (SH-W) vs. Wait List. Figure A.7. IC-W vs. Self-Help (SH). Figure A.8. GSH-W vs. SH. Figure A.9. SH-W vs. SH. Figure A.10. IC-W vs. MC-W. Figure A.11. IC-W vs. GSH-W. Figure A.12. MC-W vs. GSH-W. Figure A.13. MC-W vs. SH-W. Figure A.14. GSH-W vs. SH-W. Figure A.15. Inconsistency plot. Table A.1. Pairwise treatment comparisons for network meta-analysis for weight loss. Figure A.16. Comparison adjusted funnel-plot. [file 40337_2021_432_MOESM1_ESM.pdf]

Additional file

Figure A.1. Graphic for risk of bias

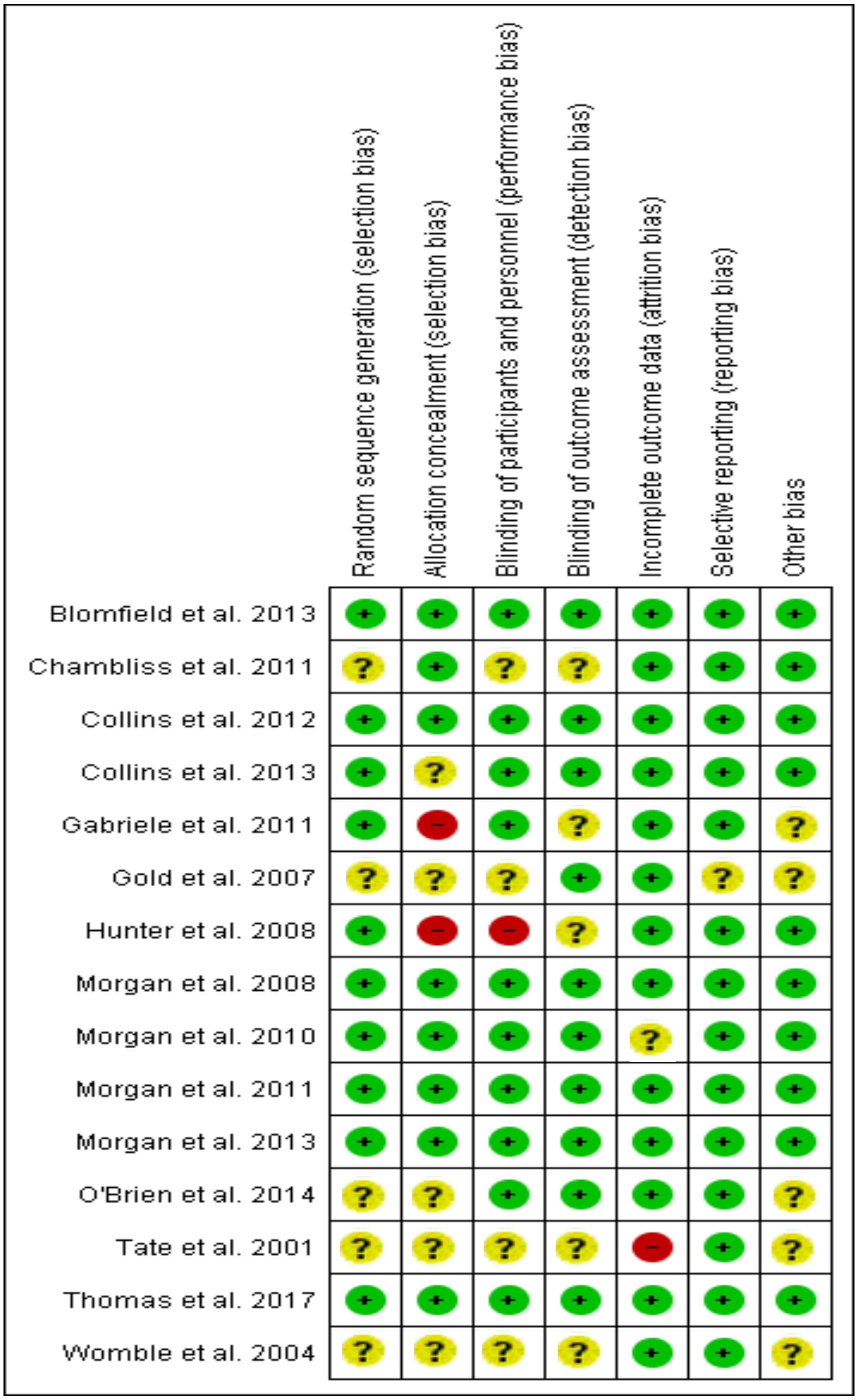

Figure A.2. Contribution direct comparisons table to the network meta-analysis

Network meta-analysis estimates

	Direct comparisons in the network												
	AvsB	AvsC	AvsE	AvsF	BvsC	BvsD	BvsF	CvsD	CvsE	CvsF	DvsE	DvsF	EvsF
Mixed estimates													
AvsB	12.8	3.2	12.9	17.3	5.1	10.8	17.5	1.1	3.1		3.4	6.3	6.4
AvsC	8.2	7.5	16.0	15.0	11.8	0.5	4.1	7.0	11.1	9.3	1.9	4.6	3.0
AvsE	6.2	3.0	44.4	14.1	1.5	3.2	1.5	0.3	5.6	0.8	4.6	1.7	13.2
AvsF	10.2	3.4	17.3	27.2	1.5	2.4	9.4	1.3	2.5	3.0	3.2	6.9	11.7
BvsC	5.4	4.9	3.3	2.8	19.2	12.9	15.2	9.2	9.0	10.5	1.8	2.0	3.9
BvsD	4.3	0.1	2.6	1.6	4.9	38.5	18.8	4.0	0.5	0.4	3.4	20.6	0.2
BvsF	6.7	0.6	1.2	6.1	5.5	17.9	35.1	0.1	1.6	3.4	1.3	16.6	4.1
CvsD	1.9	4.6	1.2	3.9	14.6	16.9	0.3	11.8	9.1	10.4	4.3	17.5	3.6
CvsE	4.0	5.6	14.9	5.4	11.0	1.7	5.3	6.9	15.3	8.9	5.1	3.5	12.3
CvsF		5.5	2.4	7.9	15.2	1.6	13.6	9.4	10.6	13.7	0.8	11.8	7.5
DvsE	5.2	1.1	14.4	8.1	2.5	12.8	5.0	3.8	6.0	0.8	8.2	18.0	14.1
DvsF	2.4	0.7	1.4	4.5	0.7	19.9	16.7	4.0	1.0	3.0	4.6	36.8	4.3
EvsF	4.7	0.9	20.0	14.5	2.7	0.4	7.8	1.6	7.0	3.6	6.8	8.0	22.2
Indirect estimates													
AvsD	9.1	3.0	13.7	17.1	1.6	14.8	4.1	3.6	2.5	0.3	5.3	19.1	5.8
BvsE	7.9	1.1	16.1	7.1	5.7	12.0	17.1	1.2	6.4	0.5	6.0	4.7	14.3
**Entire network**	6.1	3.1	12.2	10.3	7.1	10.7	10.9	4.5	6.3	4.6	4.2	11.5	8.6
**Included studies**	3	1	5	3	2	3	3	1	2	1	1	2	2

A = Intensive Contact Web; B = Minimal Contact Web; C = Guided Self-Help Web; D = Self-Help Web; E = Self-Help; F = Wait list.

* Results expressed as percentages.

Figure A.3. Intensive Contact Web (IC-W) vs. Wait list

	Experimental			Control				Mean Difference
Study or Subgroup	Mean	SD	Total	Mean	SD	Total	Weight	IV, Random, 95% CI
Blomfield et al. 2013	-5.4	4.1	53	-0.6	2.6	52	33.2%	-4.80 [-6.11, -3.49]
Morgan et al. 2011	-4	4.5	65	0.3	2.7	45	31.3%	-4.30 [-5.65, -2.95]
Morgan et al. 2013	-4.4	4.3	53	-0.5	1.9	52	35.5%	-3.90 [-5.17, -2.63]
**Total (95% CI)**			**171**			**149**	**100.0%**	**-4.32 [-5.08, -3.57]**

Heterogeneity: Tau² = 0.00; Chi² = 0.94, df = 2 (P = 0.63); I² = 0%
Test for overall effect: Z = 11.22 (P < 0.00001)

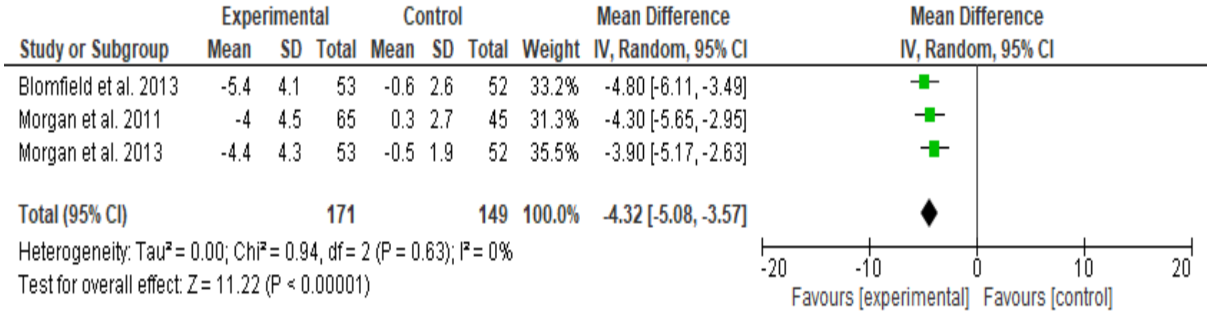

Figure A.4. Minimal Contact Web (MC-W) vs. Wait list

	Experimental			Control				Mean Difference
Study or Subgroup	Mean	SD	Total	Mean	SD	Total	Weight	IV, Random, 95% CI
Chambliss et al. 2011	-2.45	3.05	45	0.3	2.23	30	22.4%	-2.75 [-3.95, -1.55]
Collins et al. 2012	-2.98	4.05	106	0.36	2.33	104	40.2%	-3.34 [-4.23, -2.45]
O'Brien et al. 2014	-3	4	98	0.4	2.4	97	37.4%	-3.40 [-4.32, -2.48]
**Total (95% CI)**			**249**			**231**	**100.0%**	**-3.23 [-3.80, -2.66]**

Heterogeneity: Tau² = 0.00; Chi² = 0.81, df = 2 (P = 0.67); I² = 0%
Test for overall effect: Z = 11.19 (P < 0.00001)

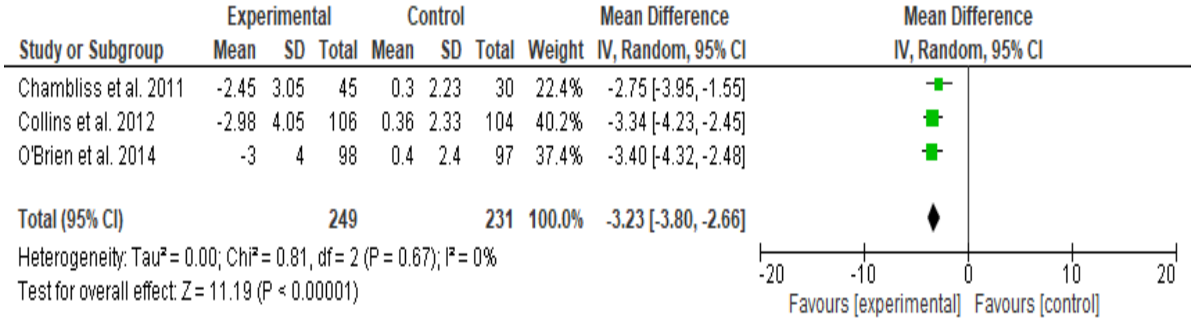

Figure A.5. Guided Self-Help Web (GSH-W) vs. Wait List

	Experimental			Control				Mean Difference
Study or Subgroup	Mean	SD	Total	Mean	SD	Total	Weight	IV, Random, 95% CI
Chambliss et al. 2011	-2.72	3.32	45	0.3	2.23	30	100.0%	-3.02 [-4.28, -1.76]
**Total (95% CI)**			**45**			**30**	**100.0%**	**-3.02 [-4.28, -1.76]**

Heterogeneity: Not applicable
Test for overall effect: Z = 4.71 (P < 0.00001)

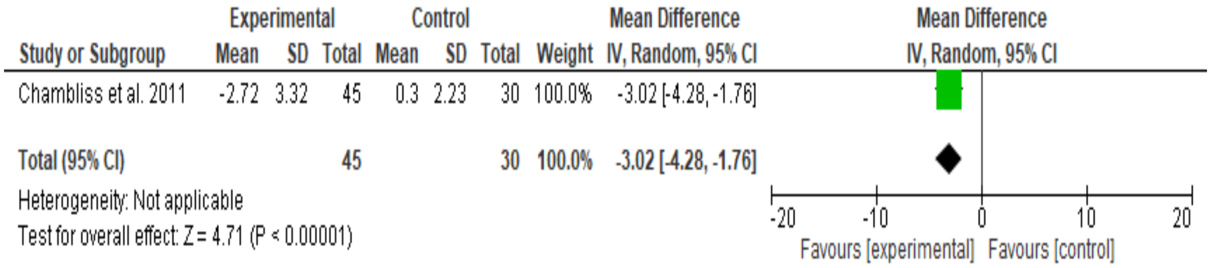

Figure A.6. Self-Help Web (SH-W) vs. Wait List

Study or Subgroup	Experimental Mean	Experimental SD	Experimental Total	Control Mean	Control SD	Control Total	Weight	Mean Difference IV, Random, 95% CI
Collins et al. 2012	-2.14	3.32	99	0.36	2.33	104	52.7%	-2.50 [-3.29, -1.71]
O'Brien et al. 2014	-2.2	3.4	94	0.4	2.4	97	47.3%	-2.60 [-3.44, -1.76]
**Total (95% CI)**			**193**			**201**	**100.0%**	**-2.55 [-3.12, -1.97]**

Heterogeneity: Tau² = 0.00; Chi² = 0.03, df = 1 (P = 0.86); I² = 0%
Test for overall effect: Z = 8.68 (P < 0.00001)

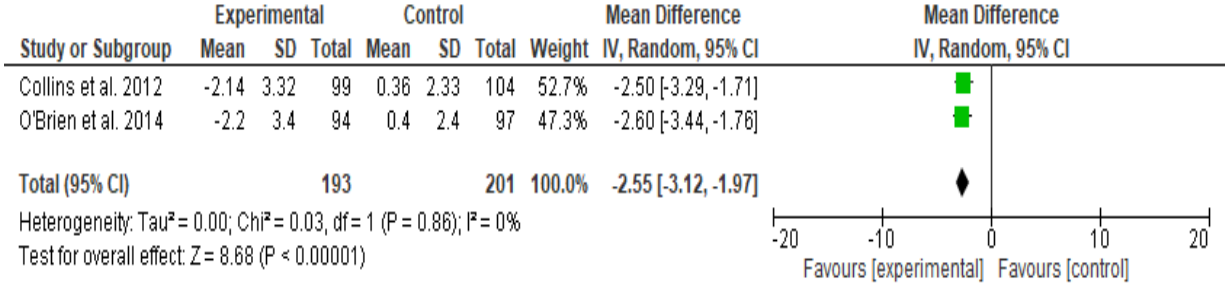

Figure A.7. IC-W vs. Self-Help (SH)

Study or Subgroup	Experimental Mean	Experimental SD	Experimental Total	Control Mean	Control SD	Control Total	Weight	Mean Difference IV, Random, 95% CI
Blomfield et al. 2013	-5.4	4.1	53	-3.8	3.9	54	13.1%	-1.60 [-3.12, -0.08]
Hunter et al. 2008	-1.3	4.1	224	0.6	3.4	222	61.8%	-1.90 [-2.60, -1.20]
Morgan et al. 2008	-4.8	4.6	34	-3	4.4	31	6.3%	-1.80 [-3.99, 0.39]
Morgan et al. 2010	-4.8	4.6	34	-3	4.4	31	6.3%	-1.80 [-3.99, 0.39]
Morgan et al. 2013	-4.4	4.27	53	-3	3.9	54	12.5%	-1.40 [-2.95, 0.15]
**Total (95% CI)**			**398**			**392**	**100.0%**	**-1.79 [-2.33, -1.24]**

Heterogeneity: Tau² = 0.00; Chi² = 0.40, df = 4 (P = 0.98); I² = 0%
Test for overall effect: Z = 6.37 (P < 0.00001)

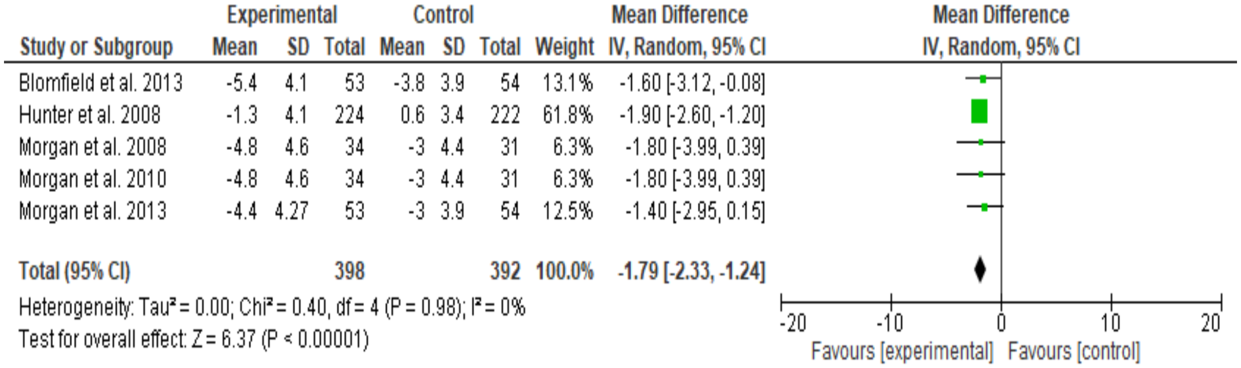

Figure A.8. GSH-W vs. SH

Study or Subgroup	Experimental Mean	Experimental SD	Experimental Total	Control Mean	Control SD	Control Total	Weight	Mean Difference IV, Fixed, 95% CI
Thomas et al. 2017	-1.6	4.9	91	-1.3	5	86	69.5%	-0.30 [-1.76, 1.16]
Womble et al. 2004	-0.8	3.6	23	-3.3	4.1	24	30.5%	2.50 [0.30, 4.70]
**Total (95% CI)**			**114**			**110**	**100.0%**	**0.55 [-0.66, 1.77]**

Heterogeneity: Chi² = 4.31, df = 1 (P = 0.04); I² = 77%
Test for overall effect: Z = 0.89 (P = 0.37)

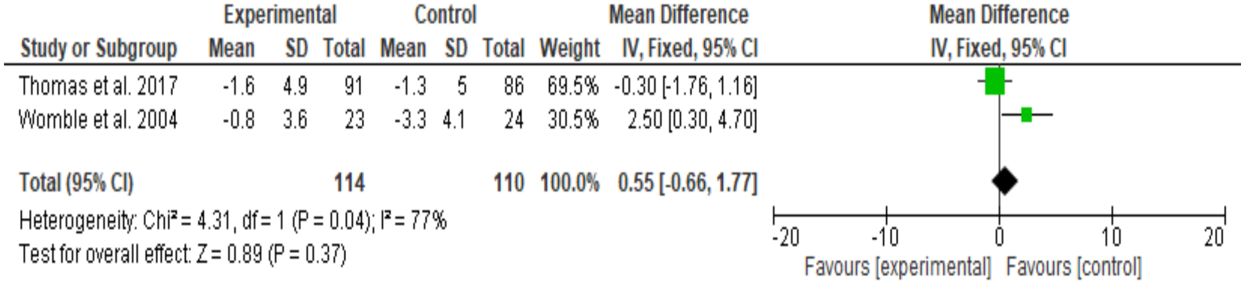

Figure A.9. SH-W vs. SH

Study or Subgroup	Experimental Mean	Experimental SD	Experimental Total	Control Mean	Control SD	Control Total	Weight	Mean Difference IV, Random, 95% CI
Thomas et al. 2017	-2.1	4.6	94	-1.3	5	86	100.0%	-0.80 [-2.21, 0.61]
**Total (95% CI)**			**94**			**86**	**100.0%**	**-0.80 [-2.21, 0.61]**

Heterogeneity: Not applicable
Test for overall effect: Z = 1.11 (P = 0.27)

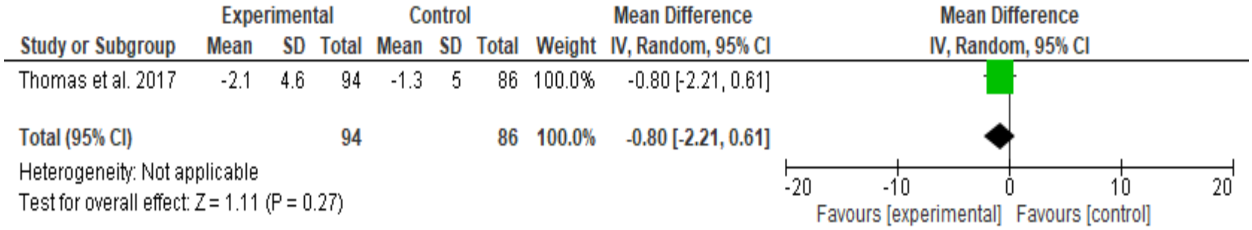

Figure A.10. IC-W vs. MC-W

Study or Subgroup	Experimental Mean	Experimental SD	Experimental Total	Control Mean	Control SD	Control Total	Weight	Mean Difference IV, Random, 95% CI
Gabriele et al. 2011	-4.34	4.04	35	-3.31	4.01	35	34.3%	-1.03 [-2.92, 0.86]
Gold et al. 2007	-6.8	7.8	62	-3.3	5.8	62	23.4%	-3.50 [-5.92, -1.08]
Tate et al. 2001	-4.1	4.5	46	-1.6	3.3	45	42.3%	-2.50 [-4.12, -0.88]
**Total (95% CI)**			**143**			**142**	**100.0%**	**-2.23 [-3.53, -0.93]**

Heterogeneity: Tau² = 0.35; Chi² = 2.72, df = 2 (P = 0.26); I² = 26%
Test for overall effect: Z = 3.37 (P = 0.0008)

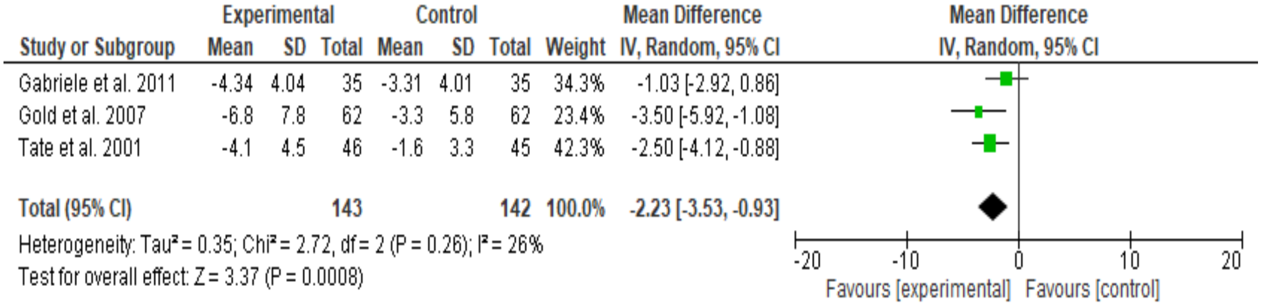

Figure A.11. IC-W vs. GSH-W

Study or Subgroup	Experimental Mean	Experimental SD	Experimental Total	Control Mean	Control SD	Control Total	Weight	Mean Difference IV, Random, 95% CI
Gabriele et al. 2011	-4.34	4.04	35	-3.06	3.38	34	100.0%	-1.28 [-3.04, 0.48]
**Total (95% CI)**			**35**			**34**	**100.0%**	**-1.28 [-3.04, 0.48]**

Heterogeneity: Not applicable
Test for overall effect: Z = 1.43 (P = 0.15)

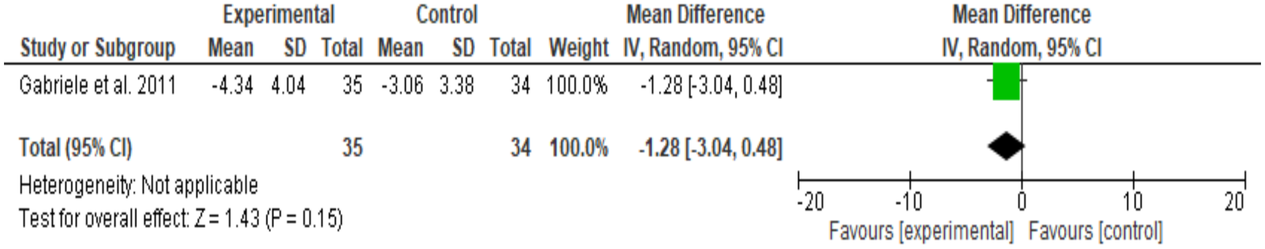

Figure A.12. MC-W vs. GSH-W

Study or Subgroup	Experimental Mean	Experimental SD	Experimental Total	Control Mean	Control SD	Control Total	Weight	Mean Difference IV, Fixed, 95% CI
Chambliss et al. 2011	-2.45	3.05	45	-2.72	3.32	45	63.8%	0.27 [-1.05, 1.59]
Gabriele et al. 2011	-3.31	4.01	35	-3.06	3.38	34	36.2%	-0.25 [-2.00, 1.50]
**Total (95% CI)**			**80**			**79**	**100.0%**	**0.08 [-0.97, 1.13]**

Heterogeneity: Chi² = 0.22, df = 1 (P = 0.64); I² = 0%
Test for overall effect: Z = 0.15 (P = 0.88)

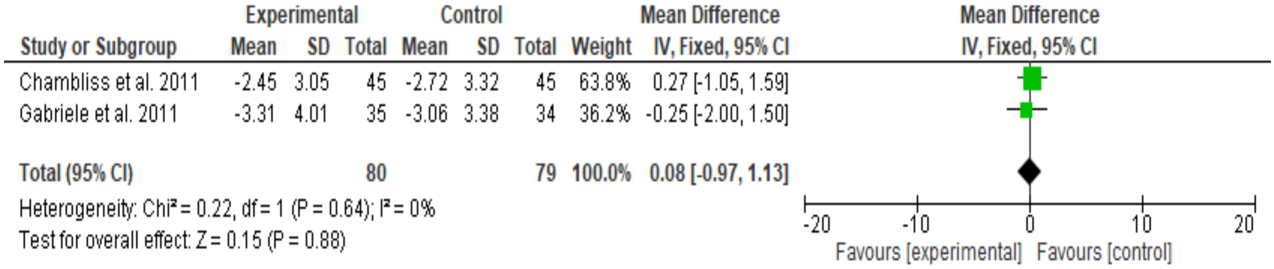

Figure A.13. MC-W vs. SH-W

Study or Subgroup	Experimental Mean	Experimental SD	Experimental Total	Control Mean	Control SD	Control Total	Weight	Mean Difference IV, Random, 95% CI
Collins et al. 2012	-2.98	4.05	106	-2.14	3.32	99	32.9%	-0.84 [-1.85, 0.17]
Collins et al. 2013	-3.3	4.5	158	-2.7	4	143	36.5%	-0.60 [-1.56, 0.36]
O'Brien et al. 2014	-3	4	98	-2.2	3.4	94	30.6%	-0.80 [-1.85, 0.25]
**Total (95% CI)**			**362**			**336**	**100.0%**	**-0.74 [-1.32, -0.16]**

Heterogeneity: Tau² = 0.00; Chi² = 0.13, df = 2 (P = 0.94); I² = 0%
Test for overall effect: Z = 2.50 (P = 0.01)

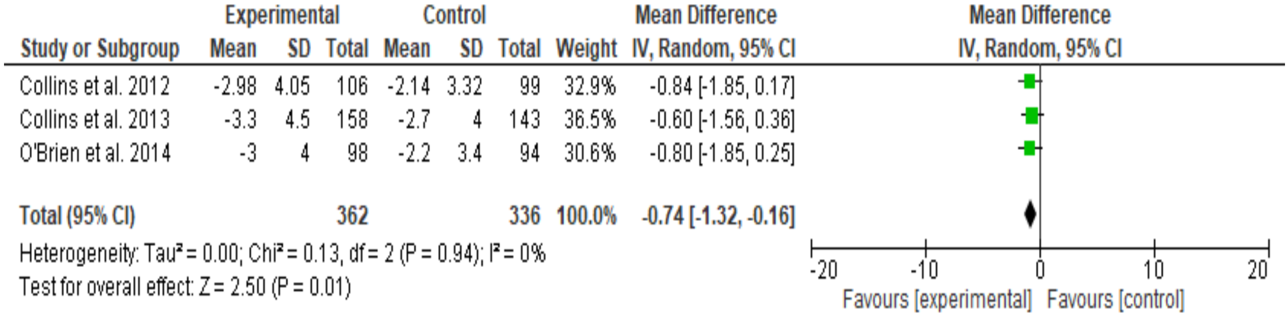

Figure A.14. GSH-W vs. SH-W

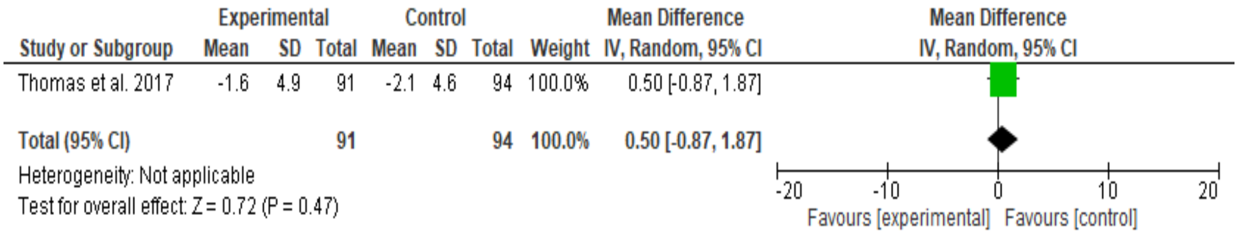

Figure A.15. Inconsistency plot

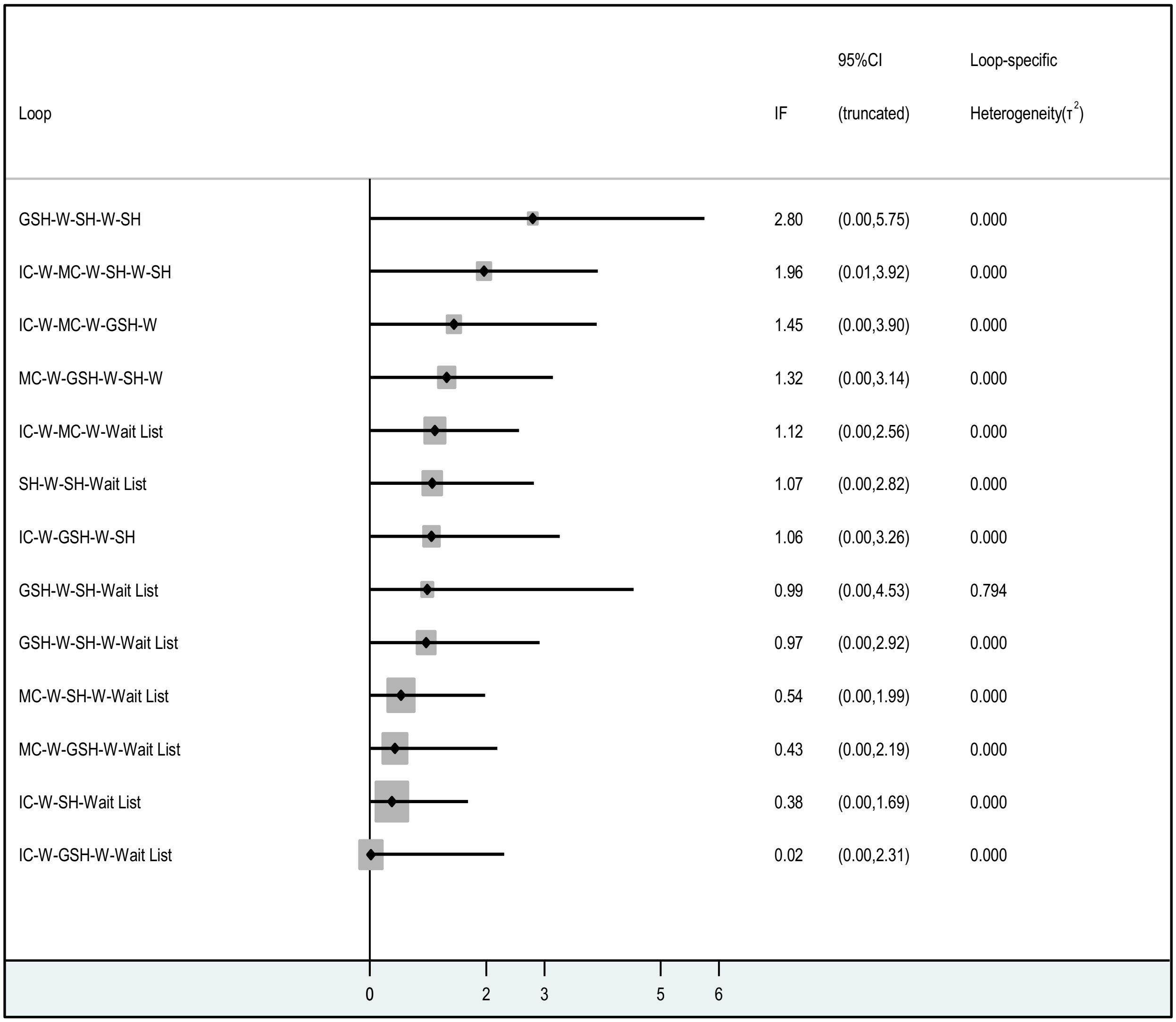

CI: Confidence Interval; GSH-W: Guided Self-Help Web; IC-W: Intensive Contact Web; IF: Inconsistency factor; MC-W: Minimal Contact Web; SH: Self-Help; SH-W: Self-Help Web.

Table A.1. Pairwise treatment comparisons for network meta-analysis for weight loss

**IC-W**	0.01	**-4.31**	-1.78	-1.25	**-1.86**
	(-1.61, 1.64)	**(-5.22, -3.41)**	(-4.10, 0.54)	(-3.26, 0.77)	**(-3.61, -0.12)**
-0.01	**MC-C**	**2.81**	1.28	**3.41**	**1.51**
(-1.64, 1.61)		**(1.46, 4.15)**	(-0.48, 3.04)	**(1.76, 5.06)**	**(0.43, 2.60)**
**4.31**	**-2.81**	**GSH-W**	-1.53	0.60	-1.30
**(3.41, 5.22)**	**(-4.15, -1.46)**		(-3.74, 0.68)	(-0.36, 1.56)	(-3.03, 0.43)
1.78	-1.28	1.53	**SH-W**	2.13	0.23
(-0.54, 4.10)	(-3.04, 0.48)	(-0.68, 3.74)		(-0.28, 4.54)	(-1.83, 2.29)
1.25	**-3.41**	-0.60	-2.13	**SH**	-1.90
(-0.77, 3.26)	**(-5.06, -1.76)**	(-1.56, 0.36)	(-4.54, 0.28)		(-3.87, 0.08)
**1.86**	**-1.51**	1.30	-0.23	1.90	**Wait List**
**(0.12, 3.61)**	**(-2.60, -0.43)**	(-0.43, 3.03)	(-2.29, 1.83)	(-0.08, 3.87)	

GSH-W: Guided Self-Help Web; IC-W: Intensive Contact Web; MC-W: Minimal Contact Web; SH: Self-Help; SH-W: Self-Help Web.

Figure A.16. Comparison adjusted funnel-plot

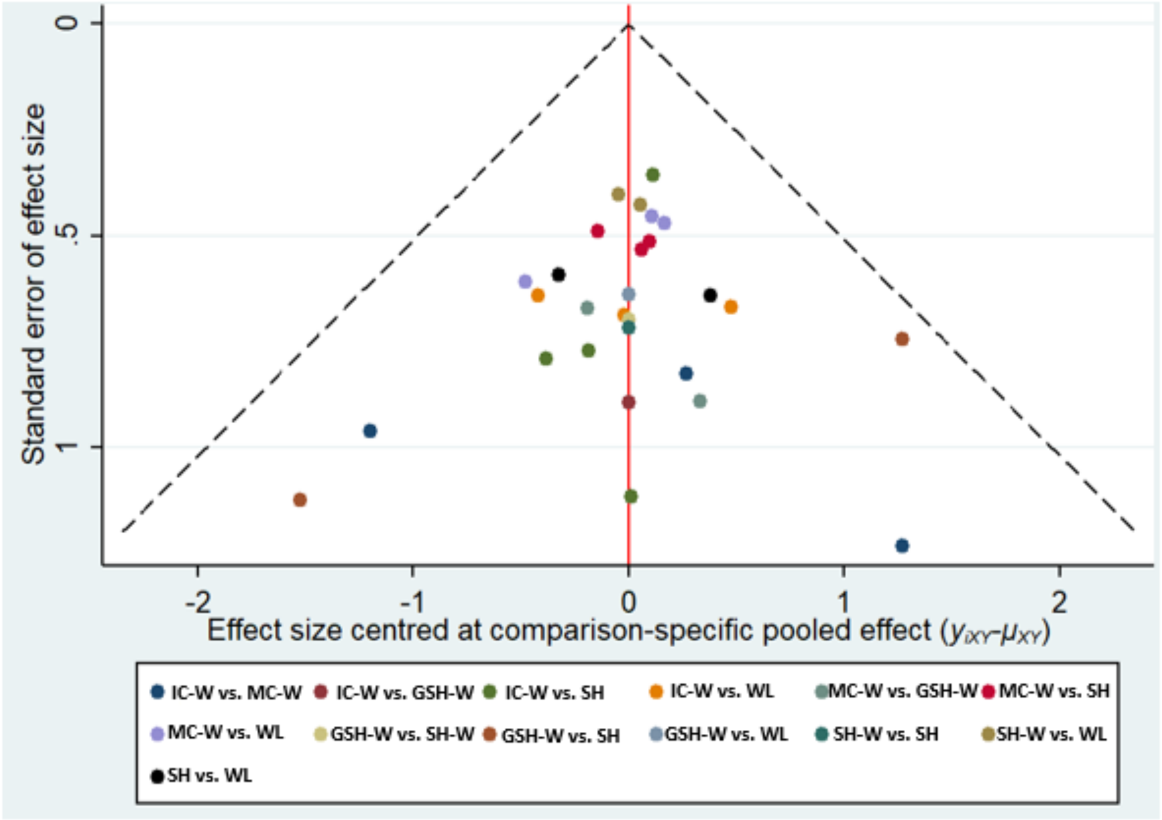

CI: Confidence Interval; GSH-W: Guided Self-Help Web; IC-W: Intensive Contact Web; IF: Inconsistency factor; MC-W: Minimal Contact Web; SH: Self-Help; SH-W: Self-Help Web; WL: Wait List.
